# Glucocorticoid-Induced Osteoporosis: Pathogenesis, the Impact of Different Administration Routes on Bone Mineral Density, and Fracture Risk and Treatment Options—A Narrative Review

**DOI:** 10.3390/jcm15072488

**Published:** 2026-03-24

**Authors:** Monika Kapszewicz, Marta Michalska-Kasiczak, Ewa Sewerynek

**Affiliations:** Department of Endocrine Disorders and Bone Metabolism, Medical University of Lodz, 90-752 Lodz, Poland

**Keywords:** glucocorticoid-induced osteoporosis, bone mineral density, fracture risk, routes of administration, osteoporosis treatment

## Abstract

Glucocorticoids (GCs) are widely used for their potent anti-inflammatory and immunosuppressive effects, but their use is strongly associated with negative impacts on bone health. Rapid bone loss and an increased risk of fragility fractures are characteristics of glucocorticoid-induced osteoporosis (GIOP), the most common type of secondary osteoporosis. While oral GCs are a well-known cause of GIOP, growing evidence suggests that non-oral routes of administration may also negatively affect the skeleton. This review summarizes current knowledge on the pathophysiology of GIOP, highlighting the complex relationship between direct and indirect mechanisms. It examines the effects of various routes of GC administration—oral, intravenous, inhaled, topical, and epidural—on bone mineral density, microarchitecture, and fracture. While parenteral GCs may have fewer systemic effects than oral therapy, long-term exposure or high cumulative doses may still cause clinically significant skeletal deterioration. This review also discusses current methods for assessing, preventing, and treating the fracture risk associated with GIOP. These strategies include lifestyle modifications, calcium and vitamin D supplements, and medications such as denosumab, bisphosphonates, and anabolic agents. Reducing the incidence of glucocorticoid-associated fractures and improving prevention and treatment requires an understanding of how GCs impact bone.

## 1. Introduction

Glucocorticoids (GCs) are a widely recognized class of pharmacological agents known for their anti-inflammatory and immunosuppressive effects across medical disciplines. Their administration is associated with several side effects, including the deterioration of glycemic control and the onset of diabetes [1]. Additionally, evidence indicates that there is a correlation between GCs and psychiatric disorders, thrombotic complications, embolism, elevated liver function parameters, and osteoporosis [2].

Glucocorticoid-induced osteoporosis (GIOP) is a serious clinical problem and accounts for an estimated 20% of all cases of secondary osteoporosis. About 30–50% of patients treated with long-term GCs develop fragility fractures, resulting in a significant reduction in their quality of life [3]. During the first 3 to 6 months of GC therapy, there is a rapid initial loss of 3–27% of bone mineral density (BMD). Bone loss predominantly affects trabecular bone, particularly in the lumbar spine [4].

In a study by Cheng et al., significantly lower BMD in L1–L4 of the spine and a higher FRAX risk of major and hip fractures were observed in patients treated with low-dose GCs (2.5–7.5 mg/day oral prednisolone or equivalent dose) compared to controls [5]. The effects of daily and cumulative oral GC doses on the risk of fracture were assessed in a study including 244.235 GCs users and an equal number of controls. The results demonstrated a dose-dependent increase in vertebral fracture risk of 1.55 at <2.5 mg/day, 2.59 at prednisolone doses of 2.5–7.5 mg and to 5.18 at doses of ≥7.5 mg [6].

Although oral GCs have a significant association with GIOP, other forms of therapy, such as topical, intravenous, inhalation, or epidural therapies, may also contribute to bone loss and fracture risk, especially with prolonged and high-dose exposure [7,8,9,10,11,12].

The purpose of this review is to evaluate the impact of GCs treatment on BMD and fracture risk, particularly when GCs are administered through non-oral routes. We also assess the effectiveness of therapeutic and preventive strategies for managing GC-induced bone loss, aiming to provide clinically relevant recommendations for the prevention and management of GIOP across diverse patient populations.

## 2. Literature Search Strategy

A literature search was conducted in PubMed/MEDLINE, Scopus, and Web of Science for articles published between 1983 and 2025. The search included combinations of the keywords “glucocorticoid-induced osteoporosis”, “glucocorticoids”, “corticosteroids”, “bone mineral density”, “fracture risk”, “bone metabolism”, “systemic glucocorticoids”, “inhaled corticosteroids”, “topical corticosteroids”, and “epidural steroid injections”, as well as terms related to the treatment of glucocorticoid-induced osteoporosis (e.g., “bisphosphonates”, “denosumab”, “teriparatide”, and “romosozumab”). Original studies, systematic reviews, and meta-analyses published in English and addressing the effects of GCs on bone health were included. Reference lists of relevant articles were also screened to identify additional studies.

## 3. Mechanisms of Glucocorticoid Effects on Bone

**Figure 1:**
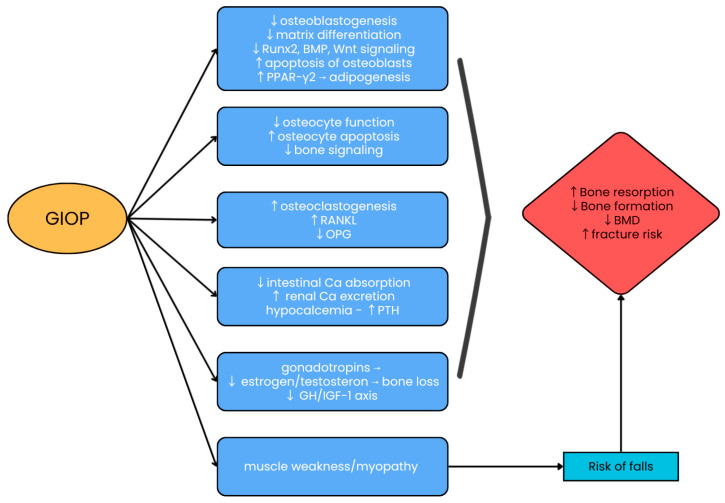
Main mechanisms of glucocorticoid-induced osteoporosis [4,13,14]. ↑—increase; ↓—decrease; Runx2—Runt-Related Transcription Factor 2; BMPs—Bone Morphogenetic Proteins; Wnt—Wingless and Int-1; PPAR—Peroxisome Proliferator–Activated receptors; RANKL—Receptor Activator for Nuclear Factor κB Ligand; OPG—Osteoprotegerin; Ca—Calcium; PTH—Parathyroid hormone; GH—Growth hormone; IGF-1—Insulin-Like Growth Factor type 1.

The pathogenesis of GIOP is highly complex, involving both direct and indirect effects on bone tissue (Figure 1).

### 3.1. Inhibition of Osteoblast Function

GCs directly affect bone metabolism by inhibiting mesenchymal stem cell differentiation into osteoblasts and suppressing osteoblast precursor proliferation by arresting the cell cycle in the G1 phase [15]. This is due to decreased expression of cell cycle activators, such as cyclin A, cyclin D, CDK2, CDK4, and CDK6, and increased expression of cell cycle inhibitors, such as p53, p21, and p27 [15,16]. Additionally, GCs interfere with several signaling pathways in osteoblasts, including the bone morphogenetic protein (BMP)-Runx2, Wnt, and peroxisome proliferator-activated receptor-γ2 (PPAR-γ2) pathways, promoting differentiation of mesenchymal progenitor cells toward adipocytes rather than osteoblasts, thereby reducing osteoblastogenesis [4,16].

Therapeutic doses of GCs have been shown to reduce bone formation markers and anabolic osteoblast functions, such as osteocalcin, alkaline phosphatase, and N-terminal procollagen I propetide (P1NP), leading to a decrease in bone matrix production and a subsequent reduction in bone mass and quality [16]. Excess GCs induce apoptosis of osteoblasts and osteocytes, resulting in compromised bone formation and mechanical strength independent of BMD loss [14].

Moreover, GCs suppress bone formation by reducing insulin-like growth factor type 1 (IGF-1) through altering the expression of IGF-binding proteins (IGFBPs), including decreased IGFBP-3 and IGFBP-5 and increased expression of IGFBP-4 [17,18].

### 3.2. Promotion of Osteoclast Activity

Increased bone resorption in GIOP is associated with receptor activator of nuclear factor κB (NF-κB) ligand (RANKL) and osteoprotegerin (OPG) [19]. RANKL is secreted by osteoblasts and precursor cells, binds to the RANK receptor on osteoclasts, and stimulates their differentiation and activity. OPG inhibits bone resorption by blocking RANKL-RANK interaction [20]. GCs stimulate osteocytes to increase sclerostin production, thereby elevating the RANKL/OPG ratio, which promotes osteoclast differentiation and decreases osteoclast apoptosis, thereby promoting bone resorption [21].

### 3.3. Impaired Calcium Homeostasis

GCs impair intestinal calcium absorption by decreasing the expression of active duodenal calcium transporters independently of suppressing 25-hydroxycholecalciferol. Furthermore, they also enhance urinary calcium excretion by inhibiting calcium reabsorption in the renal tubules [22]. Most studies do not support the thesis that GCs directly increase serum parathyroid hormone (PTH) levels [23]. However, GCs increase skeletal sensitivity to PTH, potentially by increasing the expression and availability of PTH receptors on osteoblasts, which may exacerbate the catabolic effects on bone [24]. However, chronic GC treatment alters PTH secretion dynamics, decreasing tonic and increasing pulsatile release, which may reflect compensatory mechanisms to counteract GC-induced bone loss [25]. Distinct skeletal patterns differentiate hyperparathyroidism and GIOP: excess PTH results in a preferential loss of bone mass in the cortical skeleton with relative preservation of trabecular skeleton [26]. Conversely, in GIOP, the pattern is reversed: GCs primarily cause trabecular bone loss, leading to a significant reduction in lumbar spine density [27].

### 3.4. Effects of Hormonal Regulation

By inhibiting the hypothalamic–pituitary–gonadal axis, GCs reduce estrogen and testosterone levels, which leads to an increased the risk of osteoporosis [28]. Estrogen deficiency affects the expression of estrogen target genes and increases the secretion of proinflammatory cytokines (IL-1, IL-6, TNF-alfa), enhances osteoclastogenesis, and accelerates bone loss [28,29]. In postmenopausal women undergoing regular GC therapy for asthma, hormone replacement therapy (HRT) has been found to be equally effective as etidronate, a bisphosphonate, in preventing BMD loss over a five-year period. HRT has been shown to be comparable to etidronate, with statistically significant results observed only at the proximal femur [30]. In another study, 21 patients with rheumatoid arthritis treated with GCs and receiving HRT had significant increases in lumbar spine BMD (3.75%) and in hip BMD (by 1.62%) [31]. However, the use of HRT is limited by the elevated risk of breast cancer, cardiovascular disease, cerebrovascular accidents, and thromboembolic events, as demonstrated in the Women’s Health Initiative trial [32]. Although HRT may offer benefits in terms of bone health, its overall risk profile should be carefully considered, especially in postmenopausal women undergoing GC therapy [32,33].

Low testosterone levels in men also increase the risk of osteoporosis. Testosterone deficiency enhances bone resorption by stimulating RANKL-mediated osteoclast activity [34]. In a randomized controlled trial of 51 men receiving long-term GC therapy, participants were given testosterone, nandrolone decanoate, or placebo for 12 months. Only testosterone treatment resulted in a significant increase in their lumbar spine BMD (mean 4.7 ± 1.1% (*p* < 0.01)), with no significant change in hip or total body BMD in the nandrolone or in either group [35]. Testosterone therapy appears to be most beneficial in hypogonadal men, in whom it improves BMD and alleviates symptoms of testosterone deficiency [34,36]. In eugonadal men, the impact on BMD may be less significant. Its use should be carefully considered based on the patient’s individual needs and risk profile [35].

GCs suppress growth hormone (GH) secretion and inhibit its anabolic effects on skeletal muscle. GH influences bone metabolism by direct osteoblast stimulation and indirect mechanisms involving IGF-1 production in the liver and peripheral tissues. GH or IGF-1 deficiency impairs osteoblastogenesis, reduces bone strength, and increases fracture risk [37]. Conversely, GH excess disrupts bone structure through increased remodeling and trabecular perforations [38]. Short-term administration of recombinant human GH has been shown to significantly increase bone remodeling in GC-exposed patients, suggesting potential therapeutic value in GIOP [39]. However, its long-term efficacy, safety, and cost-effectiveness require further research before widespread clinical use.

### 3.5. Induction of Myopathy

In the hip and shoulder areas, GCs induce muscle weakness and skeletal muscle loss, resulting in myopathy due to enhanced myofibril proteolysis [40]. GCs upregulate myostatin, a negative regulator of muscle hypertrophy. In mouse models, deletion of the myostatin gene prevents GC-induced myofibril proteolysis and muscle loss [41]. Muscle changes can affect up to 60% of GC-treated patients and may develop within 7 days of initiating treatment [40,42]. The negative effects of GCs on muscles can increase the risk of fractures by increasing the incidence of falls [43].

In summary, the mechanisms through which GCs exert their effects on bone tissue are multifaceted. These mechanisms include disruption of calcium homeostasis, stimulation of osteoclast activity, suppression of osteoblast function, and changes in hormonal regulation. When combined, these side effects increase the risk of osteoporosis and fractures in patients receiving GC treatment.

## 4. Impact of Non-Oral Glucocorticoid Administration Routes on Bone Health Outcomes

### 4.1. Intravenous Administration

High-dose intravenous therapy is a potent immunosuppressive treatment that is used across many medical specialties, such as rheumatology, pulmonology, dermatology, and endocrinology [7,44]. If GCs are administered intravenously rather than orally, systemic adverse effects, such as weight gain, Cushingoid obesity, diabetes, and hypertension, are minimized, providing potential benefits for patients receiving pulse therapy [45]. In fact, there is conflicting evidence about the effects of intravenous GCs on bone tissue. Intravenous GCs may reduce some of the risks associated with osteoporosis, bone metabolism, and BMD [7,46]. A study in patients with Graves ophthalmopathy showed that intravenous methylprednisolone treatment decreased markers of bone formation (osteocalcin, procollagen I carboxyterminal propeptide (PICP)) and increased markers of bone resorption (urinary deoxypyridinoline (DPD) and Ca excretion) [47]. High-dose intravenous GC therapy rapidly suppresses bone formation and transiently increases bone resorption, as shown by changes in BTMs in patients with multiple sclerosis who were receiving short-term pulse therapy [48]. In one study, a cumulative dose of methylprednisolone exceeding 7.5 mg daily of prednisone suggested that intravenous GCs can cause significant disturbances in bone metabolism [49]. Analysis of the effects of a 12-week course of intravenous high-dose methylprednisolone in patients with Graves ophthalmopathy revealed that bone turnover and cortical porosity at the radius and tibia decreased significantly by −7.67 ± 3.13% (*p* = 0.04) and −3.30 ± 2.17% (*p* = 0.04) during treatment. BMD remained stable at 12 weeks but increased at 24 weeks at the femoral neck (+2.26 ± 3.61%, *p* < 0.01) and at the total hip (+2.24 ± 4.24%, *p* = 0.02) [50]. A study on multiple sclerosis patients treated with high pulses of intravenous GCs confirmed that there was no change in BMD after 6 months of therapy [48]. A pilot study demonstrated a significant 2.4% decrease in the mean trabecular bone score (TBS) of the lumbar spine compared to the baseline (*p* < 0.05), indicating that trabecular bone microarchitecture may be more sensitive to GC effects than BMD [51].

These findings highlight that intravenous GCs modulate bone effects differently from oral therapy, with site-specific impacts and dose-dependent risks. Monitoring trabecular microarchitecture and prioritizing underlying disease management (e.g., thyroid function) may mitigate bone health risks in long-term therapy.

### 4.2. Inhaled Administration

Inhaled corticosteroids (ICs) are essential for treating respiratory diseases like asthma and chronic obstructive pulmonary disease (COPD), significantly reducing airway inflammation, symptoms, exacerbations and mortality in asthma [52]. ICs are primarily recommended for COPD patients with elevated blood eosinophil counts (>300 cells/µL) and frequent exacerbations (≥2/year), since they reduce mortality and exacerbation rates [53].

After inhalation, approximately 40–90% of the GC dose is deposited in the mouth or pharynx and swallowed, with partial first-pass metabolism, preventing systemic exposure. Pulmonary absorption significantly contributes to systemic bioavailability [54]. Although ICs often provide clinical benefits, they also can cause systemic adverse effects, including osteoporosis, pneumonia, cataracts, glaucoma, and adrenal suppression [55]. A recent meta-analysis found that higher doses of ICs (>120 mg/year) increased osteoporotic fractures (OR = 1.19; 95%CI: 1.05–1.35) and osteoporosis (OR = 1.63; 95%CI: 1.33–1.99), despite there being no significant reduction in BMD of the lumbar spine and femur in asthmatics receiving ICs compared to non-ICs. These results suggest that IC-treated patients may be more susceptible to fractures at greater BMD levels [56]. In contrast, other reviews report no negative effects on fracture risk or BMD in asthmatic and COPD patients treated with ICs [12,57]. A meta-analysis of 13 studies revealed contradictory findings. While one study reported no change in BMD, several others demonstrated a decrease in BMD among IC-treated patients. Remarkably, only one study showed a higher incidence of fractures without an adequate decline in BMD [11]. A small cross-sectional study in children reported a significant reduction in lumbar spine BMD (*p* < 0.005), which may have affected peak bone mass and future osteoporosis risk [58,59].

ICs are essential for managing asthma and COPD; however, evidence regarding their effects on bone health is inconsistent. While some studies suggest that higher cumulative doses may increase fracture risk, particularly with long-term use, many others report no significant effects on BMD or fracture incidence. The available evidence indicates that any potential adverse effects on bone are likely modest, dose-dependent, and influenced by additional osteoporosis risk factors. Further studies are needed to resolve these inconsistencies and to optimize the risk–benefit balance in chronic respiratory disease management.

### 4.3. Topical Administration

Topical corticosteroids (TCs), although primarily intended for regional treatment in dermatological diseases such as psoriasis or atopic dermatitis, can exert adverse effects on bone, particularly with prolonged or high-dose use [60]. The systemic effects of TCs are influenced by several factors, including the total skin area treated, the frequency of application, the amount and potency of the steroid ointment, the duration of therapy, and the integrity of the skin barrier. The skin barrier is compromised by inflammatory skin conditions, which makes it easier for GCs to penetrate the skin and increase their systemic bioavailability [8]. A large retrospective cohort study in Denmark, including 723,251 individuals, demonstrated a significant dose–response relationship between the use of potent or very potent TCs and an increased risk of osteoporosis and major osteoporotic fractures. The risk of fractures increased markedly with a dose of ≥500 g/year (mometasone equivalent) and continued to grow steadily. The use of TCs was found to account for 2.7% (95% CI, 1.7–3.8%) of osteoporotic fractures and 4.3% (95% CI, 2.7–5.8%) of osteoporosis cases in the study population [9]. Another large, nationwide case–control study similarly found that higher cumulative TC doses over a 5-year period were associated with an increased risk of osteoporosis and osteoporotic fractures [61]. The investigations concluded a correlation between increased risk of osteoporosis and major osteoporotic fractures and higher cumulative doses of TCs. In high-risk groups receiving long-term or high-dose TC therapy, it is recommended to measure BMD and fracture risk. Additionally, for patients who need long-term, high-intensity treatment, the use of other topical anti-inflammatory and immunosuppressive medications is recommended [9,61].

### 4.4. Epidural Administration

Epidural steroid injections (ESIs) are commonly used to treat radicular pain in patients with intervertebral disc pathology or nerve root irritation due to lumbar spinal stenosis, particularly when conservative therapies have failed, and patients are not suitable candidates for immediate neurosurgical intervention [62]. The most used GCs for ESIs include methylprednisolone, triamcinolone, betamethasone, and dexamethasone, which are administered with or without a local anesthetic and are injected into the epidural space. A systematic review revealed that ESIs were associated with significantly decreased BMD in four out of six included studies [63]. One study reported that repeated ESIs (approximately 14 injections with a cumulative triamcinolone dose of 400 mg) led to a significant decrease in lumbar spine BMD over a period of 2 years [10].

In contrast, another study found no significant relationship between the cumulative methylprednisolone dose and BMD at the lumbar spine, femoral neck, and total hip in patients treated with ESIs [64]. In the prospective study, ESIs led to an increase in serum C-telopeptide of collagen I (CTX), a marker of bone resorption, which persisted for three months after injection. Serum procollagen type 1 N-terminal propeptide (P1NP), a marker of bone formation, showed a delayed increase after three months of follow-up. BMD at the spine and femoral neck decreased significantly three months after ESI, while total hip BMD remained unchanged. After six months, 20.17% of patients showed a moderate or significant decrease in BMD. The data indicated high bone turnover and formation, and these effects were further confirmed after six months of follow-up. At this point, all bone turnover markers (BTMs) returned to near-baseline values, and BMD also improved [65].

Overall, evidence on the effects of ESIs on bone health remains limited and inconsistent. Some studies suggest that repeated injections and higher cumulative GC doses may be associated with transient reductions in BMD and increased bone turnover, whereas others report no significant effects. Further well-designed longitudinal studies are needed to clarify their long-term impact on bone metabolism and fracture risk.

## 5. Treatment of Glucocorticoid-Induced Osteoporosis

The guidelines and recommendations make no distinction between routes of GC administration other than oral therapy. Therefore, the guidelines for GIOP related to oral GC users should form the basis for the treatment plan, prevention, and monitoring strategies in patients receiving non-oral GCs. The implementation of preventive and therapeutic approaches should be based on factors such as age, cumulative GC dose, fracture history, and baseline BMD. Regular monitoring and patient education are essential to optimize bone health and reduce the risk of GIOP [33,66,67].

### 5.1. Prevention Strategies

A balanced diet providing adequate essential nutrients is a critical component of bone health during GIOP treatment, including total calcium intake (from both dietary sources and elemental calcium supplemented) of 1000 mg/day for men aged 50–70 years and 1200 mg/day for women ≥ 51 years and men ≥ 71 years; protein intake of 1.2 g/kg b.w./day; potassium at approx. 3500 mg/day; and magnesium at >300 mg/day [33,68]. Current guidelines from the American College of Rheumatology (ACR) recommend vitamin D 25(OH)D supplementation (800–1000 IU/day or more) for adults receiving prednisone > 2.5 mg/day for more than 3 months to maintain serum 25(OH)D levels of 30–50 ng/mL [33].

Additionally, lifestyle changes such as smoking cessation, limiting alcohol consumption to two drinks per day or less, and engaging in regular weight-bearing or resistance exercise are recommended [33].

Furthermore, the use of the lowest effective dose of GCs for the shortest duration can also help prevent GIOP adverse effects. When clinically appropriate and equally effective, alternative routes of GC administration, such as topical or intra-articular delivery, should be considered to reduce systemic exposure [67].

### 5.2. Fracture Risk Assessment and Treatment Eligibility

Fracture risk should be assessed before initiating or continuing GCs therapy (Table 1) [3,33,40]. The Fracture Risk Assessment (FRAX) tool is recommended for adults aged 40 and older. FRAX estimates the 10-year risk of hip fracture and major osteoporotic fractures (including hip, spine, distal forearm, and humerus fractures) [69]. Although FRAX includes the femoral neck, vertebral fractures are more common in GIOP [68]. In patients taking low doses of GCs (<2.5 mg/day), the FRAX of a major osteoporotic fracture is decreased by about 20% and requires dose adjustment. For medium doses (2.5–7.5 mg/day), no correction is needed. For high doses (>7.5 mg/day), FRAX results should be increased by 15% for MOF and 20% for hip fracture, as recommended by the ACR [33,68].

Early BMD changes in GC therapy can be subtle and are detectable by dual-energy X-ray absorptiometry (DXA) [33,67]. Before initiating GC therapy, spine BMD, along with vertebral fracture assessment (VFA) or P-A and lateral spine X-rays, should be obtained for all patients over 40 years of age [33]. It is essential to differentiate spinal deformities (e.g., Schmorl’s nodes or Scheuermann’s disease) from vertebral fractures on standard X-rays. For patients aged <40 years taking GC ≥ 2.5 mg/day with one or more osteoporosis risk factors, BMD with VFA should be measured promptly before initiating therapy [33].

The European Calcified Tissue Society (ECTS) recommends assessing fall risk using patient history or conducting a test such as the Timed Up and Go Test [66]. Based on BMD and FRAX results, patients are stratified into low-, moderate-, and high-risk fracture groups. The latest ACR guidelines have introduced a very-high-risk group, which includes patients with previous osteoporotic fractures, very low BMD, very high FRAX risk, or those receiving high daily (≥30 mg/day for >30 days) or cumulative doses of GC (≥5 g/year) [33].

The Polish osteoporosis guidelines are mostly in line with international standards from the ACR, the International Osteoporosis Foundation (IOF) and ECTS [33,66,67,70]. They focus on early risk assessment, suggest bisphosphonate prevention in adults ≥50 years receiving prednisone ≥ 5 mg/day for more than 3 months if they are at risk of fracture, and mandate prevention in those aged ≥65 years. Use of teriparatide in very high-risk cases, when available, reflects the ACR principles, with broader acceptance of raloxifene in postmenopausal women. Overall, these recommendations reflect evidence-based, internationally harmonized approaches to GIOP management [67].

### 5.3. Treatment Option

**Figure 2:**
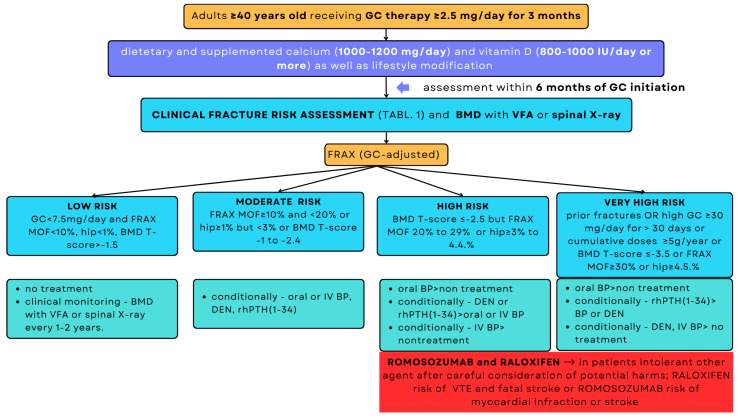
Strategy for assessing fracture risk and initiating treatment in adults ≥40 years old [33]. MOF—major osteoporotic fracture; FRAX-fracture risk assessment tool; BP—bisphosphonate; IV—intravenous; rhPTH(1–34)—teriparatide; DEN—denosumab; VTE—Venous Thromboembolism.

**Figure 3:**
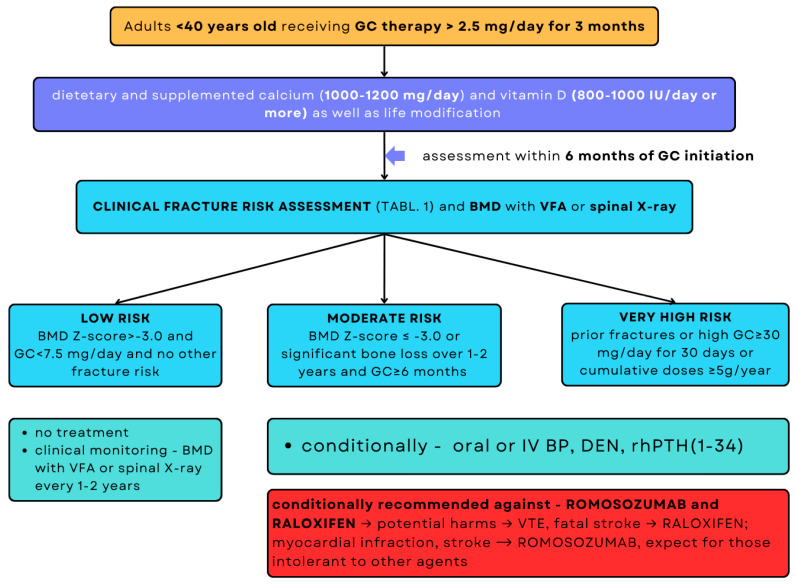
Strategy for assessing fracture risk and initiating treatment in adults <40 years old [33].

The therapeutic strategy for patients taking prednisone (or equivalent GC) at a dose of ≥2.5 mg/day for >3 months depends on the fracture risk, BMD and VFA or spine X-ray, FRAX score, and age based on the ACR guidelines (Figure 2 and Figure 3) [33].

#### 5.3.1. Bisphosphonates

Bisphosphonates (BPs) are the main antiresorptive therapy in GIOP [33]. They inhibit the expression of osteoclast-dependent cytokines such as IL-6 and TNF-alpha, as well as farnesyl pyrophosphate synthase, thereby causing osteoclast apoptosis and inhibiting bone resorption [71]. The current guidelines suggest the use of alendronate (70 mg/week orally), risedronate (35 mg/week orally or 75 mg for two consecutive days per month), and intravenous zoledronic acid (5 mg once a year) for the prevention and treatment of GIOP in both men and women [33,66,67]. There is strong evidence supporting the efficacy of these BPs in increasing BMD at the lumbar spine and hip and reducing vertebral fracture risk [72,73]. Zoledronic acid has shown superior effectiveness compared to risedronate in enhancing lumbar spine BMD for the prevention and treatment of GIOP [74,75]. Zoledronic acid offers the benefit of less frequent administration, potentially enhancing patient compliance compared to daily oral medications such as risedronate [74]. Although BPs are generally well tolerated, oral BPs can cause gastrointestinal side effects, while zoledronic acid can cause transient post-injection symptoms such as fever or myalgia [76]. Long-term therapy with rare but serious side effects, such as osteonecrosis of the jaw (incidence 1/10,000 to 100,000 person-years) and atypical femur fractures (incidence 3.2–50/100,000 person-years), can be associated with BPs use [77]. Premenopausal women should be closely monitored when BP is prescribed, as the potential accumulation of these substances in the placental barrier can have adverse effects on a fetus [78].

Treatment duration is typically 5 years for oral formulations and 3 years for intravenous zoledronate, followed by reassessment of fracture risk and treatment safety [68,79,80]. For adults aged ≥40 years with GIO, treatment duration may need to be extended, especially for those on high doses of GCs or for patients who have suffered fragility fractures. The ACR recommends continuing oral BPs treatment for more than 5 years or switching to intravenous BPs if there are concerns about adherence or absorption or considering alternative drug classes [33]. A “drug holiday” of 2 to 3 years may be considered in patients who have been treated for more than 5 years, particularly if they are at lower fracture risk. During this period, patients should be monitored for changes in BTM and BMD. Treatment may be resumed if fracture risk increases or if significant BMD loss occurs [68,79,80].

#### 5.3.2. Denosumab

Denosumab is a monoclonal antibody directed against RANKL. By binding to RANKL, denosumab inhibits its interaction with the RANK receptor on osteoclasts, thereby reducing osteoclast activity and bone resorption [81]. A systemic review demonstrated that denosumab significantly increased BMD compared with BPs, with a mean lumbar spine BMD gain of 2.32% and hip BMD gain of 1.52% [82]. Furthermore, denosumab showed both non-inferiority and superiority to risedronate over 12 months in improving BMD at the lumbar spine in patients undergoing GCs treatment (*p* < 0.0001) [83]. This monoclonal antibody decreased BTM, such as CTX and P1NP, within 3 months, demonstrating its efficacy for early intervention in GIOP, where bone loss occurs rapidly [84]. Even though there is considerable evidence supporting the beneficial effect of denosumab on BMD, the available studies on fracture risk reduction compared with BPs remain limited. Nevertheless, denosumab provides a similar level of protection against vertebral fractures [85,86].

The ACR guideline recommends denosumab administration (60 mg subcutaneously every 6 months) for patients at moderate-to-high fracture risk without a predefined maximum duration. Long-term use may be suitable for patients with high fracture risk, but careful monitoring is essential. Sequential therapy with BPs is advised after discontinuing denosumab to prevent rebound bone loss [33].

#### 5.3.3. Teriparatide

Endogenous PTH, consisting of 84 amino acids, is a major regulator of calcium and phosphate metabolism in bone tissue and the kidneys. Teriparatide (rhPTH(1–34)), a recombinant human PTH peptide, contains the active N-terminal fragment of endogenous PTH and promotes osteoblastogenesis and osteoblast differentiation, exerting anabolic effects on bone mass [87]. It was the first anabolic agent to be approved by the Food and Drug Administration (FDA) for the treatment of osteoporosis in postmenopausal women and men with an increased risk of fractures [88]. It is currently recommended for the treatment of GIOP, especially in patients at very high risk of fractures. Teriparatide is considered as an initial treatment in patients with high fracture risk and a FRAX-adjusted score ≥ 20% or a T-score ≤ −2.5 with suboptimal response to BPs or contraindications or intolerance to antiresorptive therapy [33,66,67].

In an 18-month randomized, double-blind trial, in patients with osteoporosis receiving long-term GCs, teriparatide showed a more significant increase in lumbar spine BMD (7.2% vs. 3.4%, *p* < 0.001), and hip BMD compared to the alendronate group. Fewer new vertebral fractures occurred in the teriparatide group (0.6% vs. 6.1%, *p* < 0.005), while the incidence of non-vertebral fractures was similar in both groups (5.6% vs. 3.7%, *p* = 0.36) [89]. A randomized controlled trial found that teriparatide was superior to risedronate in increasing BMD after 18 months in men with GIOP (16.3% vs. 3.8%, *p* < 0.005), with a nonsignificant reduction in clinical fractures [90]. In 2024, a Bayesian network meta-analysis involving 11 randomized controlled trials established that teriparatide and denosumab were more successful in increasing BMD at the lumbar spine and femur neck, and in decreasing vertebral fractures. Teriparatide exhibited superior efficacy compared with BPs in increasing lumbar spine BMD (+7.2% vs. +3.4% at 36 months) and reducing vertebral fractures (1.7% vs. 7.7% incidence) [91]. Research findings suggest that teriparatide is a preferred initial anabolic intervention for enhancing BMD and reducing the risk of fractures in patients diagnosed with severe GIOP who are at a substantial risk of fractures or demonstrate an insufficient response to antiresorptive therapy [90,91,92].

However, its costliness compared to antiresorptive drugs, the need for daily subcutaneous administration (20 mcg/day), regular monitoring of serum calcium levels due to the risk of transient hypercalcemia, and the limitation of treatment to 24 months are notable limitations. Additionally, antiresorptive therapy should be used after teriparatide treatment to maintain and consolidate BMD gains and prevent fragility fractures [33,66].

#### 5.3.4. Romosozumab

Romosozumab is a humanized monoclonal antibody that inhibits sclerostin, a protein that negatively regulates bone metabolism. This inhibition promotes bone formation and suppresses bone resorption [93]. Treatment with romosozumab increases BMD more significantly and rapidly than alendronate or teriparatide and is superior in reducing the risk of vertebral and nonvertebral fractures in postmenopausal women with osteoporosis [94,95]. The FDA has approved romosozumab as a treatment for severe osteoporosis with a high fracture risk in postmenopausal women at a dose of 210 mg monthly (two subcutaneous injections of 105 mg) for 12 months [96]. In a murine model, romosozumab prevented GC-induced reduction in both trabecular and cortical bone mass, increasing trabecular bone volume by 60–125%, apparent bone strength of lumbar vertebrae by 30–70%, and cortical bone mass in the femur by 10–18% compared to placebo [97]. In a clinical study involving patients with rheumatic diseases initiating GC therapy, romosozumab led to the greatest increase in lumbar spine BMD (+8.6%) after 12 months compared to denosumab (+3.3%) or risedronate (−0.4%) [98]. In a pilot randomized controlled trial involving high-risk patients receiving oral prednisolone (≥5 mg/day), romosozumab showed a greater increase in lumbar spine BMD than denosumab (+7.3% vs. +2.3%, *p* < 0.001), with comparable improvements in hip BMD [99]. Romosozumab may increase the risk of myocardial infarction and cardiovascular death and should be used with caution in patients with cardiovascular disease [93]. These findings indicate that romosozumab could be an effective treatment option for patients with GIOP, particularly those at high fracture risk or those who have demonstrated an inadequate response to prior therapy.

However, romosozumab is not yet approved by the FDA specifically for GIOP, and additional research is needed to clarify its role in this setting [33,66]. The ACR guidelines conditionally recommend romosozumab over no treatment for patients at high or very high risk of fracture [33].

### 5.4. Monitoring

It is essential to prioritize monitoring and follow-up in patients diagnosed with GIOP to encourage adherence to preventive measures, assess treatment effectiveness, and review fracture risk. The DXA is central to BMD monitoring, and vertebral fracture assessment should be considered within one year of initiating GCs therapy. The follow-up intervals should be individualized based on patient-specific risk factors [33,66,67].

TBS can be an additional tool for monitoring GIOP, as it provides insight into bone microarchitecture. Expert opinion of European Society for Clinical and Economic Aspects of Osteoporosis, Osteoarthritis and Musculoskeletal Diseases (ESCEO), and the IOF suggests that TBS enhances fracture risk prediction in both primary and secondary osteoporosis and may offer valuable information when used alongside BMD and FRAX to support decisions regarding osteoporosis treatment. TBS has also been shown to assist in monitoring responses to therapies such as denosumab and anabolic agents. Nevertheless, current evidence does not support modifying treatment solely on the basis of TBS findings [66,100]. BTMs can be considered for monitoring the effects of antiresorption or anabolic therapy in GIOP [66]. When GCs therapy is discontinued, it is strongly recommended to reassess fracture risk to determine whether GIOP preventive treatment remains necessary [33,66,67]. Table 2 compares the main points of assessment, management, treatment, and monitoring based on the ACR, ECTS, Belgian Bone Club, and Polish guidelines.

## 6. Conclusions

GIOP is a serious and underdiagnosed clinical problem, especially for individuals receiving long-term GC treatment. BMD testing is performed on less than 10% of GC-treated patients, and less than 22% receive bone-active therapy [49]. Fracture risk increases depending on the dose and duration of treatment. While oral GCs are the most widely studied in relation to GIOP, non-oral GCs also contribute to bone loss and fracture risk, especially with prolonged or high-dose exposure. Due to the common use of GCs in clinical practice, it is vital to promptly diagnose and treat GIOP. Preventive strategies, including calcium and vitamin D supplementation, weight-bearing physical activities, and smoking cessation, should be combined with pharmacological therapies.

This study highlights the importance of considering the route of GC administration when assessing the risk of GIOP. Moreover, it underscores the significance of comprehensive care approaches, including both preventative and therapeutic interventions, to lessen the effects of GCs therapy, particularly in individuals exposed to long-term or high-dose GCs therapy.

## Figures and Tables

**Table 1:** Clinical risk factors for glucocorticoid-induced osteoporosis [3,33,40].

High doses (≥2.5 mg/day or ≥5 mg/day of prednisolone or equivalent) and/or long-term GCs use (≥3 months)
Older adults (≥65 years)
Postmenopausal women
Underlying diseases (hypogonadism, hyperparathyroidism, thyroid diseases, rheumatoid arthritis, malabsorption, chronic liver diseases, asthma/COPD)
Calcium and vitamin D deficiencies
Sedentary lifestyle
Smoking history
Alcohol consumption
Low body weight or BMI
Family history of osteoporosis, hip fractures
Personal history of fragility fractures
Low baseline BMD
Use of anticonvulsants, proton pump inhibitors, SSRIs, or aromatase inhibitors

**Table 2:** Comparison of the main points of assessment, management, treatment and monitoring based on the ACR, ECTS, Belgian Bone Club and Polish guidelines [33,66,67,70].

	ACR (2022) [33]	ECTS (2024) [70]	Polish Guideline (2023) [67]	Belgian Bone Club (2022) [66]
Population threshold for treatment	Adults (≥40 years)	Adults (≥40 years)	Adults (≥50 years)	Adults (≥40 years)
GC dose threshold (prednisone or equivalent)	2.5 mg/day ≥ 3 months	2.5 mg/day ≥ 3 months	5 mg/day ≥ 3 months	5 mg/day ≥ 3 months
Risk assessment tools	FRAX (GC-adjusted), DXA, vertebral imaging	FRAX (GC dose-adjusted), DXA	FRAX, DXA, vertebral assessment; national fracture risk charts	FRAX, TBS, DXA
Treatment threshold	FRAX risk (MOF ≥ 10–20% or Hip > 1%), T-score < –1.0 or prior fragility fracture	FRAX risk MOF ≥ 10% or T-score < –1.5	High or very high fracture risk, FRAX risk MOF ≥ 10% or Hip ≥ 3%, or T-score ≤ –1.5	FRAX risk MOF ≥ 10% or T-score ≤ –1.5
First-line therapy	Bisphosphonates (alendronate, risedronate)	Bisphosphonates	Bisphosphonates; consider early anabolic therapy in severe GIOP	Bisphosphonates
Alternative/second-line	Denosumab, teriparatide, i.v. zoledronate	Teriparatide, denosumab, romosozumab	Teriparatide, denosumab for very high fracture risk	Teriparatide, denosumab
Calcium/Vitamin D supplementation	1000–1200 mg Ca, 800–1000 IU Vit D/day	1000–1200 mg Ca, 800–1000 IU Vit D/day	1000–1200 mg Ca + 1000–2000 IU Vit D/day	1000–1200 mg Ca, 800–1000 IU Vit D/day
Monitoring	DXA every 1–2 years	DXA every 2 years	DXA every 1–2 years; reassess risk yearly	DXA every 1–2 years

## Data Availability

The data presented in this study are available on request from the corresponding author.
